# Hypertrophic Cardiomyopathy in Underrepresented Populations: Clinical and Genetic Landscape Based on a Russian Single-Center Cohort Study

**DOI:** 10.3390/genes14112042

**Published:** 2023-11-04

**Authors:** Olga S. Chumakova, Tatiana N. Baklanova, Natalia V. Milovanova, Dmitry A. Zateyshchikov

**Affiliations:** 1 Moscow Healthcare Department, City Clinical Hospital 17, 119620 Moscow, Russia; gkb17@zdrav.mos.ru (T.N.B.); zateyschikovda@zdrav.mos.ru (D.A.Z.); 2 E.I. Chazov National Medical Research Center for Cardiology, 121552 Moscow, Russia; 3 Research Centre for Medical Genetics, 115522 Moscow, Russia; poltavets69@yandex.ru

**Keywords:** hypertrophic cardiomyopathy, Russian, Slavic, underrepresented population, specific characteristics, genetics, *MYBPC3*, *MYH7*, *TPM1*, *FLNC*

## Abstract

Hypertrophic cardiomyopathy (HCM) is a common inherited cardiac disorder characterized by marked clinical and genetic heterogeneity. Ethnic groups underrepresented in studies may have distinctive characteristics. We sought to evaluate the clinical and genetic landscape of Russian HCM patients. A total of 193 patients (52% male; 95% Eastern Slavic origin; median age 56 years) were clinically evaluated, including genetic testing, and prospectively followed to document outcomes. As a result, 48% had obstructive HCM, 25% had HCM in family, 21% were asymptomatic, and 68% had comorbidities. During 2.8 years of follow-up, the all-cause mortality rate was 2.86%/year. A total of 5.7% received an implantable cardioverter-defibrillator (ICD), and 21% had septal reduction therapy. A sequencing analysis of 176 probands identified 64 causative variants in 66 patients (38%); recurrent variants were MYBPC3 p.Q1233* (8), *MYBPC3 p.R346H* (2), *MYH7 p.A729P* (2), *TPM1 p.Q210R* (3), and *FLNC p.H1834Y* (2); 10 were multiple variant carriers (5.7%); 5 had non-sarcomeric HCM, *ALPK3*, *TRIM63*, and *FLNC*. Thin filament variant carriers had a worse prognosis for heart failure (HR = 7.9, *p* = 0.007). In conclusion, in the Russian HCM population, the low use of ICD and relatively high mortality should be noted by clinicians; some distinct recurrent variants are suspected to have a founder effect; and family studies on some rare variants enriched worldwide knowledge in HCM.

## 1. Introduction

Hypertrophic cardiomyopathy (HCM) is the most common inherited cardiac disorder with a general population incidence of 1:200–1:500 [1,2]. HCM is defined by an increase of left ventricular (LV) wall thickness that cannot be explained solely by loading conditions; it is generally asymmetric and leads to LV outflow tract obstruction (LVOTO) in two-thirds of cases [3]. Metabolic (e.g., Anderson–Fabry or glycogen storage diseases), infiltrative (amyloidosis), mitochondrial, neuromuscular (e.g., Friedreich’s ataxia), endocrine (e.g., acromegaly) disorders and malformation syndromes (e.g., Noonan) that can mimic HCM should also be ruled out [3]. The clinical course of HCM is highly variable: some patients die suddenly at a young age, while others remain asymptomatic and have a normal lifespan, or develop HCM-associated adverse events such as progressive heart failure (HF), atrial fibrillation (AF), and embolic stroke [4]. Such clinical individual differences in HCM could be considered as due to its genetic heterogeneity, although the role of comorbidities is also being investigated [5,6]. Approximately one-third of genotyped patients carry pathogenic (P) or likely pathogenic (LP) variants (formerly mutations) in genes encoding proteins of the sarcomere [7], a contractile apparatus of cardiomyocytes, and are considered to have a Mendelian form of HCM. Several non-sarcomeric genes have also been shown to cause a Mendelian form of HCM [8]. The pathogenesis of genotype-negative HCM, which has a more benign clinical course, particularly in individuals without a family history of HCM [9], remains elusive, and a polygenic mechanism of development has been proposed [6,10]. For over 50 years of research on HCM, the vast majority of studies have concentrated on North America and Western Europe, and have included individuals of predominantly European ancestry. This is an issue that may be one of the barriers to understanding the genetics of HCM, and may lead to disparities in the effectiveness of clinical genetics applications between different populations [11,12]. Despite the success of the clinical management of HCM patients in Western countries [13], it remains a burden on healthcare systems. In other parts of the world, especially in countries with developing economies and healthcare systems where many other priorities dominate resources, there is a significant lack of systematized data on the clinical and genetic spectrum and management efficacy of relatively less common genetic disorders such as HCM, resulting in low attention to this disease [14,15,16].

The aim of this study was to evaluate clinical and genetic traits of HCM and the implementation of current diagnostic and treatment approaches in Russian patients compared to other populations.

## 2. Materials and Methods

### 2.1. Study Design

From August 2009 to December 2022, HCM patients > 16 years of age were recruited into a single-center prospective observational study at Moscow Clinical Hospital #17, Moscow, Russia. There were two sources of participants: (1) patients admitted to our hospital for various reasons (cardiological, internal medicine, or non-cardiac surgery) who had indications for echocardiography (Echo) based on complaints or unexplained changes on their electrocardiogram (ECG); and (2) HCM patients referred from other clinics to confirm their diagnosis and/or determine a treatment strategy. All candidates were re-evaluated for eligibility based on imaging criteria for HCM, namely, an increased LV wall thickness >15 mm (>13 mm for relatives) not explained solely by loading conditions. Transthoracic 2D Echo was performed by a single cardiomyopathy specialist (OSC).

### 2.2. Baseline Clinical Examination

Eligible patients provided written informed consent, followed by clinical evaluation, including physical examination, detailed personal and family history of HCM and sudden cardiac death (SCD) for at least three generations, review of available medical records for previous examinations, comorbidities and treatment, and 12-lead ECG at rest. Blood samples were collected in EDTA-containing tubes, frozen at −25 °C, and stored for further genetic testing. All patients were recommended for 24 h Holter monitoring if data for the previous year were not available. The 5-year SCD risk score was assessed using the HCM Risk-SCD model [17] in all patients with a predefined set of Echo and Holter variables. For patients enrolled before 2014, the SCD risk score was calculated retrospectively. Stress Echo was recommended for patients with no or mild LVOTO at rest or after Valsalva’s provocation. All patients were referred for contrast-enhanced cardiac magnetic resonance (CMR) imaging if there were no contraindications and if no data were available for the previous five years. Most CMR examinations were performed in a specialized imaging laboratory at Lomonosov Moscow State University. All recommended diagnostic tests were performed in assured clinics or paid for by the patients. Patients with an unclear clinical diagnosis of HCM or suspected HCM mimics were excluded. All available first-degree relatives were encouraged to undergo clinical examination, including ECG and Echo, either at our hospital or at their place of residence.

### 2.3. Genetic Testing

Genetic testing was performed in certified laboratories available at the time of patient enrollment, given financial coverage at that time. The classification of variant pathogenicity provided by the genetic laboratories was performed in accordance with the five-tier clinical significance of genetic variants (P, LP, variant of uncertain significance (VUS), likely benign (LB), and benign (B)) introduced by the joint consensus recommendation of the American College of Medical Genetics and Genomics (ACMG) and the Association for Molecular Pathology (AMP) in 2015. The ACMG/AMP guidelines include 28 criteria that are categorized by the weight and type of evidence, i.e., population frequency, type and location in the gene, segregation with phenotype, deleterious effect on the gene, or gene product functions based on functional studies or computational (in silico) analysis [18]. All primary reported variants were manually verified by the authors using the latest published reports, ClinVar entries, and the prediction tool http://franklin.genoox.com (accessed on 1 September 2023), as well as the precise location of the amino acid substitution in some encoded proteins [19,20,21]. In our study, we established two extra pathogenicity tiers, namely, VUS-favoring pathogenicity (VUS-LP) and VUS-favoring benignity (VUS-LB), to better classify VUSs with a high disease-causing potential. All VUSs, except in the *FLNC* gene, that were positioned closer to LP or LB on the pathogenicity rating scale of the AI-based interpretation tool at http://franklin.genoox.com (accessed on 1 September 2023), which provides variant-level information along with unpublished phenotype data from researchers, clinicians, and patients, were designated as VUS-LP or VUS-LB, accordingly. VUSs in the *FLNC* gene were classified as either VUS-LP or VUS-LB, depending on whether they were determined to be disease-related or neutral-benign, using a novel web-based server available at http://amiva.msp.univie.ac.at/ (accessed on 3 September 2023). This machine-learning-based neural network has achieved the highest accuracy to date of approximately 80% by utilizing in vivo and in vitro data on *FLNC* variants, along with the links between their biophysical and structural properties and disease phenotype [22].

The following genes were sequenced and analyzed in 100% of the probands: nine core sarcomeric genes encoding alpha-actin (*ACTC1*), myosin binding protein C (*MYBPC3*), myosin heavy chain 7 (*MYH7*), regulatory and essential light chains (*MYL2* and *MYL3*, respectively), troponin C (*TNNC1*), troponin I (*TNNI3*), troponin T (*TNNT2*), and alpha-tropomyosin (*TPM1*); six HCM mimic genes encoding desmin (*DES*)*,* alpha-galactosidase A (*GLA*)*,* lysosome-associated membrane protein 2 (*LAMP2*)*,* 5′-AMP-activated protein kinase subunit gamma-2 (*PRKAG2*)*,* tyrosine protein phosphatase non-receptor type 11 (*PTPN11*)*,* and transthyretin (*TTR*); and two HCM minor genes encoding filamin C (*FLNC*) and phospholamban (*PLN*). The extended panels were used as a first-line test in one-third of the probands.

### 2.4. Follow-Up

Outcomes and interventions were documented during the follow-up period through repeated clinic visits, phone calls, and the review of electronic medical records. The outcomes analyzed were the following: (1) all-cause mortality; (2) SCD; (3) new-onset AF and stroke (fatal and non-fatal); (4) HF outcome, including new HF progression and death from HF; and (5) composite outcome, including all of the above. HF progression was defined as hospitalizations requiring parenteral infusion of diuretics and/or inotropes, transition to hypokinetic HCM with a decrease in LV ejection fraction (LVEF) below 50%, or first occurrence of New York Heart Association (NYHA) class III/IV. The assessment of ventricular arrhythmias is not presented in this study. The frequency of implantable cardioverter-defibrillator (ICD) implantation and cardiac surgery was assessed over a single time period, including past history and follow-up.

### 2.5. Statistical Analysis

Continuous variables are presented as mean and standard deviation or median and interquartile range (25th–75th percentile) if not normally distributed, and compared using Student’s *t*-test and Mann–Whitney U-test. Categorical variables are presented as numbers and percentages and evaluated using chi-square or Fishers’ exact test. Time-event analysis was performed between sexes and different genotypes. Survival was evaluated by Cox proportional hazards regression. Survival curves were constructed using the Kaplan–Meier method, and comparisons were made using the log-rank test. All *p*-values were two-tailed and considered significant if <0.05. All analyses were performed with SPSS 26.0.

## 3. Results

A total of 180 unrelated probands with the HCM phenotype were recruited (88% since November 2016). In four probands (2%), genetic analysis changed the diagnosis to HCM mimics: two inherited transthyretin amyloidosis, one Fabry disease, and one desminopathy. These patients were excluded from further analysis. Cascade family screening identified an additional 17 affected relatives who were enrolled into the study. In the final analysis, a total of 193 HCM patients were included.

### 3.1. Demographic Characteristics

All participants belonged to a Caucasian race; the vast majority (*n* = 167, 87%) were from the Central region of Russia (152 from Moscow city); most Russian patients (95%) were of East Slavic origin. The males were slightly predominant (*n* = 100, 52%); the median ages at diagnosis and enrollment were 49 and 56 years, respectively; one-third of the cases were diagnosed over 60 years of age; and 34% were initially diagnosed at enrollment.

### 3.2. Clinical Characteristics

Only 21% were asymptomatic at enrollment; a quarter of the probands had a family history of HCM; the most common reason for initial diagnosis was symptoms (62%); and half of the patients had LVOTO. The clinical characteristics of the total group with respect to sex differences are summarized in Table 1.

### 3.3. Echocardiography and Electrocardiography

Resting LVOTO was seen in 30% of patients, and 19% more had a latent LVOTO; more than half of the patients had reduced LV cavity (61%) and decreased LV stroke volume (52%); the vast majority (84%) had LV diastolic dysfunction, 10% in restrictive type. In the study cohort, there were 25 apical, 5 midventricular, and 8 hypokinetic HCM. Only 2.1% of patients had a normal ECG, one in five a pathological Q wave, three-quarters negative T waves, and more than 40% a positive T wave in aVR, reflecting apical hypertrophy. Among the patients with normal ECG, there were two males and two females, aged from 36 to 57 years; three of the four were relatives first diagnosed at enrollment, and three of the four were asymptomatic; maximal LV wall thickness ranged from 13 to 16 mm; none of them had LVOTO, CAD, AF, diabetes, ICD implantation, or SRT; only one of the four had arterial hypertension, two had LV diastolic dysfunction, and one had NSVT. Holter ECG monitoring data were available in 78% of patients. Supraventricular arrhythmias were present in about 40%, and almost one-third had conduction disturbances. The main Echo, ECG, and Holter monitoring abnormalities are summarized in Table 2.

### 3.4. Genetic Testing Results

#### 3.4.1. Variant Classification

According to the primary genetic testing reports, a total of 85 rare variants were identified in 77 probands, including 19 truncating (4 frameshift, 6 splicing, and 9 nonsense) and 66 non-truncating (64 missense and 2 non-frameshift deletions). All variants were detected in heterozygosity. After reassessment of pathogenicity, 53/85 variants were classified as P/LP, 11/85 as VUS-LP, 12/85 as VUS, and 9/85 as LB or VUS-LB (Supplementary Table S1). Of the 176 genotyped probands, 66 (38%) carried a total of 64 P/LP/VUS-LP variants (some unrelated patients carried the same variant while some carried multiple variants) and were considered genotype-positive (G+) (Table 3). The remaining 110 unrelated patients (62%) were classified as genotype-negative (G−). Together with the relatives, the total G+ and G− groups consisted of 78 and 115 patients, respectively.

#### 3.4.2. Genetic Findings

**Figure 1:**
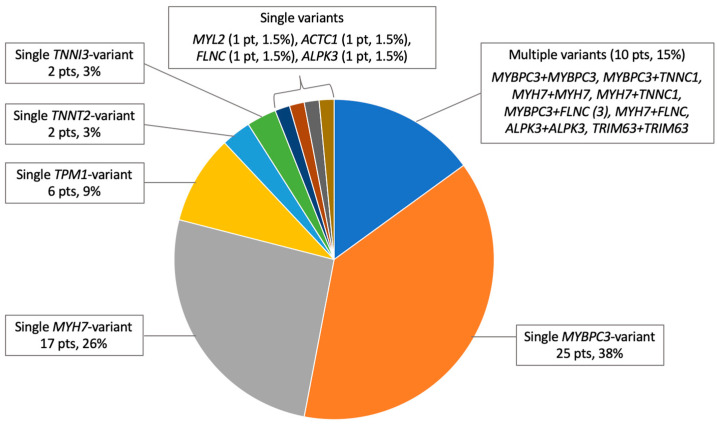
Distribution of P/LP/VUS-LP variants by gene in the cohort of unrelated G+ HCM patients (*n* = 66).

Of the 66 G+ probands, 62 patients (94%) had causative variants in sarcomeric genes and 4 (6%) in non-sarcomeric genes *FLNC* (1), *ALPK3* (2), encoding alpha-protein kinase 3 protein, and *TRIM63* (1), encoding muscle-specific RING-finger protein 1 (Table 3). Two of the most common genes were *MYBPC3* and *MYH7*, collectively accounted for 66% of P/LP/VUS-LP findings, and were responsible for the diagnosis in 50 probands, including multiple variant carriers (76% of G+ and 28% of all genotyped probands). Unique variants in *MYBPC3* (*n* = 26) included 13/22 truncating (50%) and 13/22 non-truncating (50%); all truncating and 7/13 non-truncating *MYBPC3* variants were classified as P/LP. The ratio of *MYBPC3* truncating/non-truncating variant carriers was 76/24%. The p.Q1233* variant in *MYBPC3* was found in 8 probands (12% of G+ and 4.6% of all genotyped probands). All unique variants in *MYH7* (*n* = 20) were missense. The third most common gene was *TPM1*: six probands carried four missense P/LP/VUS-LP variants, representing 9% of G+ and 3.4% of all genotyped probands. P/LP/VUS-LP unique variants in other genes were distributed as follows: *TNNT2* (2), *TNNI3* (2), *MYL2* (1), *ACTC1* (1), *TNNC1* (2), *TRIM63* (2), *ALPK3* (3), *FLNC* (4), and *LAMP2* (1). In our group, 10 patients (15% of G+ and 5.7% of all genotyped probands) carried two P/LP/VUS-LP variants in the following combinations *MYBPC3* + *MYBPC3*, *MYBPC3* + *TNNC1*, *MYH7* + *MYH7*, *MYH7* + *TNNC1*, *MYBPC3* + *FLNC* (3), *MYH7* + *FLNC*, *ALPK3* + *ALPK3*, and *TRIM63* + *TRIM63*; the patient with two variants in *TRIM63* was diagnosed with HCM as a recessive inheritance type; the cis or trans position for compound heterozygous variants was not investigated. The significant differences in genotypes between the sexes were not observed. The distribution of P/LP/VUS-LP variants in HCM-associated genes in G+ probands is shown in Figure 1.

In addition to *MYBPC3* p.Q1233*, several other variants have been reported in more than one unrelated proband: *MYBPC3 p.R346H* (2), *MYH7 p.A729P* (2), *TPM1 p.Q210R* (3), and *FLNC p.H1834Y* (2). We identified 12 novel variants, 9 of which were classified as P/LP/VUS-LP: MYBPC3 p.I324Tfs*26, MYBPC3 p.S928Hfs*124, *MYBPC3 p.C1264R*, *MYH7 p.E1388G*, *MYH7 p.N1209K*, *MYH7 p.F46Y*, *TPM1 p.K29R*, *TPM1 p.A25V*, and LAMP2 p.D82Ifs*7. The clinical characteristics of the 89 patients (77 probands and 12 relatives) carrying all 85 variants are shown in Supplementary Table S1.

#### 3.4.3. Genotype–Phenotype Correlations

- G+ versus G−

Patients with identified genetic variant(s) were younger at initial diagnosis and enrollment, significantly more likely to have familial HCM, a 5-year SCD risk score > 6%, LGE on CMR, non-compaction myocardium on Echo and/or CMR, and more likely to be diagnosed incidentally or during family screening (Table 4). G− patients were diagnosed primarily on the basis of symptoms, had a higher prevalence of resting LVOTO, LV diastolic dysfunction, comorbidities, HCM-related events, and signs of hypertrophy on ECG, and were more likely to receive beta-blocker therapy. With the exception of CMR, the frequency of diagnostic and surgical procedures was similar between the groups. The same results were obtained when comparing patients with sarcomeric variants only and G−.

- Multiple variants

There were no homozygous or compound heterozygous patients with two truncating variants (Figure 1). Compared to patients with a single P/LP/VUS-LP variant (*n* = 70), patients with two rare variants (*n* = 10) had a significantly higher SCD risk score (5.9 [3.5–7.3] vs. 2.9 [1.9–4.0], *p* = 0.011), larger left atrium (52 [43–57] vs. 43 [39–50] mm, *p* = 0.017), more frequent high systolic pulmonary artery pressure (63 vs. 14%, *p* = 0.004), and a trend toward more frequent severe NYHA class III/IV (38 vs. 11%, *p* = 0.07). The clinical course of HCM was highly variable, ranging from mild symptoms to severe HF progression and SCD in middle age. The brief clinical characteristics of all multiple variant carriers are shown in the lower part of Supplementary Table S1.

- *MYBPC3* a nd *MYH7*

Nine of sixty-six probands (12%) carried the same truncating p.Q1233* variant in *MYBPC3*. Eight of nine patients did not harbor other variants that could explain the phenotype. One proband (3B) carried another P/LP variant in *TNNC1* and was excluded from further analysis of the 1233* subgroup. Family screening revealed one additional relative with such a genotype (51Ka) (Supplementary Table S1). Compared to the other G+ patients with a single variant (*n* = 72), the p.Q1233* carriers were 7 years younger at initial diagnosis (34 [17–43] vs. 41 [30–53] years, *p* = 0.063) and 10 years younger at enrollment (38 [34–45] vs. 48 [37–61] years, *p* = 0.055); none of them had comorbidities. A comparative analysis of the 1233* subgroup and patients with other MYBPC3 single variants only (*n* = 20) and patients with MYBPC3 truncating variants only (*n* = 12) showed the same trend. Half of the p.Q1233* carriers were diagnosed with obstructive HCM, two of them underwent surgery, three patients received an ICD, one had new-onset AF, and none had hypokinetic HCM or HF progression. All patients remained alive during follow-up.

**Figure 2:**
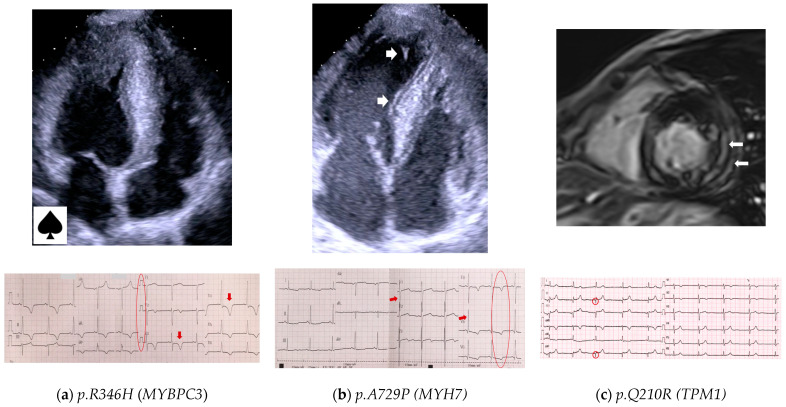
Cardiac imaging and ECG of HCM patients carrying the rare recurrent variants presented in ≥2 families in our study. (**a**) A 34-year-old male (42P) carrying the *p.R346H* variant in *MYBPC3*; Echo shows apical HCM with LV cavity in the shape of an ace of spades; ECG shows giant negative T waves (arrows) in lateral leads (Yamaguchi syndrome). (**b**) A 45-year-old female (34P), carrying the *p.A729P* variant in *MYH7*; Echo shows asymmetric septal LV hypertrophy and LV hypertrabeculation (arrows); ECG shows LV hypertrophy (Sokolow–Lyon index 45 mm pointed by arrows), and negative T waves (circle) in lateral leads. (**c**) A 38-year-old female (39Sa) carrying the *p.Q210R* variant in *TPM1*; CMR shows asymmetric LV hypertrophy with LGE (arrows); ECG shows Q-waves in leads II, aVF (circles).

The *p.R346H* variant in *MYBPC3* was found in two unrelated probands, 42P and 62B. Cascade family screening revealed two additional affected relatives (one in each family) who also carried the *p.R346H* variant (Supplementary Table S1). In one family, there were no other causative variants, and both family members (42P and 42Pa) had an apical form of HCM (Figure 2a). In another family, two siblings carried an additional variant in *ALPK3*, classified as VUS, and had septal asymmetric non-obstructive HCM. Three out of four affected individuals were asymptomatic and had no comorbidities. One 60-year-old patient (42Pa) had mild arterial hypertension and was in NYHA class II. None of the carriers of the *p.R346H* variant had any HCM-associated events in history or outcomes during follow-up.

Another variant, *p.A729P* in *MYH7*, was found in two unrelated patients (18L and 34P, Table S1). Both probands were diagnosed in middle age, were mildly symptomatic, and had non-obstructive HCM with moderate asymmetric LV septal hypertrophy of 18 mm (Figure 2b). One of the patients had two affected offspring carrying the same variant, one of whom received an ICD at the age of 15 years. All carriers of the *p.A729P* variant are alive without outcomes.

- Thin filament of sarcomere

We grouped patients carrying causative variants in thin filament sarcomeric genes (*TPM1*, *TNNT2*, *TNNI3*, *TNNC1*, and *ACTC1*) into a subgroup (*n* = 15), and compared their characteristics with other G+ patients (*n* = 63). Patients in the thin filament subgroup had a lower LV wall thickness (17 [15–20] vs. 21 [17.5–24], *p* = 0.025), and none of them had AF (0 vs. 22%, *p* = 0.044).

Six probands carried four variants in *TPM1*, and the *p.Q210R* variant was present in three unrelated patients. We performed clinical and genetic family screening in three *TPM1 p.Q210R* pedigrees. Among twelve screened relatives, six carriers were identified. None of them had other causative variants. Pediatric carriers (*n* = 3) and wild-type individuals (*n* = 6) did not show LV hypertrophy. Two out of three adult relatives carrying the *p.Q210R* variant fulfilled the criteria for HCM. Clinical analysis was therefore performed in five patients, three probands and two relatives. Mean age at diagnosis was 46 ± 14.6 years, four of five (80%) were asymptomatic, the mean maximal LV wall thickness was 17.4 ± 3.36 mm, all had an asymmetric septal pattern of LV hypertrophy, and none had LV obstruction at rest. The calculated SCD risk score was low in all patients; four carriers underwent CMR (three probands and one adolescent case), and all showed LGE (Figure 2c). Three of four also had mild hydropericardium; one proband had non-sustained ventricular tachycardia on Holter monitoring, and there was a family history of multiple SCD.

- *FLNC*

A total of nine rare variants (all missense) in *FLNC* were found; four of them were classified as VUS-LP and were detected in six patients (Supplementary Table S1); two of six were single-variant carriers, and four had additional P/LP variants in *MYBPC3* or *MYH7*. Two single-*FLNC*-variant carriers (4Ga and 28M) showed a benign clinical course.

- HCM mimic genes

Genetic testing revealed two patients with rare variants in HCM mimic genes, *LAMP2*, and *GAA*, and no other causative variants (Supplementary Table S1). None of them had clinically suspected metabolic disease, and were saved in the study as G− HCM. The p.D82Ifs*7 variant in *LAMP2* (patient 59D) was classified as LP; however, it was only identified in peripheral lymphocytes with a mosaicism level of 17%; the heart tissue was not available for molecular analysis, and it cannot be concluded with certainty that this variant is responsible for the patient’s phenotype. Patient 49D, who carried the *p.W746S* variant in *GAA*, could not be associated with recessive Pompe disease because the patient was heterozygous. In addition, another patient, 67V, carried two missense variants, initially classified as VUSs, in the *MYBPC3* and *GLA* genes. The assessment of alpha-galactosidase activity and lyso-Gb3 measurement showed no abnormalities, and the *p.I354V* variant in *GLA* was reclassified as LB.

#### 3.4.4. Outcomes

The median follow-up from enrollment was 2.8 [0.8–5.0] years. Of the 193 patients, 42 achieved the composite outcome (22%); there were 16 all-cause deaths (8%), including 5 SCD (2.6%), 3 fatal strokes (1.6%), 3 HF deaths (1.6%), and 5 other deaths (2.6%), including cancer (2), pneumonia (1), COVID-19 (1), and perioperative death (1) (Table 5). A total of 18 patients (9%) achieved the composite outcome of new-onset AF and stroke, while 15 patients (7.8%) achieved the HF outcome. Of the five patients with SCD, two had a high (>6%) and three a low (<4%) SCD risk score at enrollment. A summary of results and a comparison of mortality rates to other populations are shown in Table 5.

**Figure 3:**
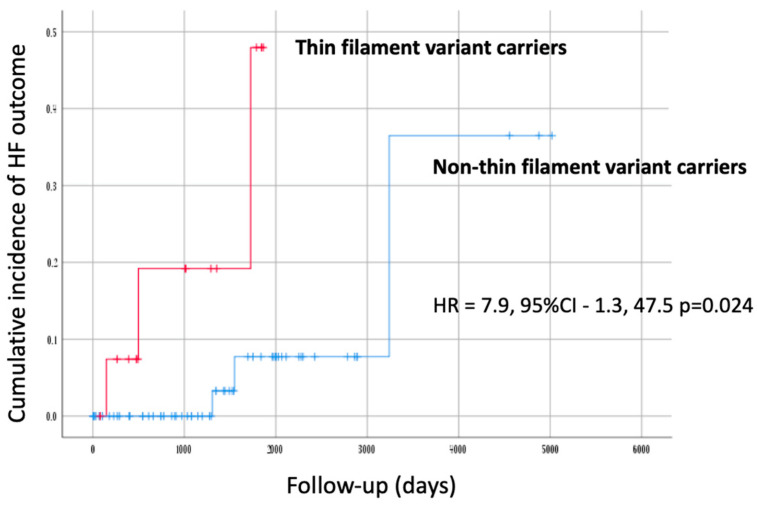
Kaplan–Meier cumulative incidence curves for HF outcome in the G+ group (*n* = 78). Patients are stratified according to the presence of a causative variant in any of the sarcomeric thin filament genes. Y-axis values indicate proportions.

The survival analysis showed a trend towards a worse prognosis for new-onset AF and stroke in female subjects, although this did not reach statistical significance (*p* = 0.055). Patients carrying thin filament variants had a significantly worse prognosis for HF (HR = 7.9, 95%CI–1.3, 47.5; *p* = 0.024) (Table 5 and Figure 3).

## 4. Discussion

Compared with the studies of the last decade [23,24,25,26,27,28], the demographic and clinical characteristics of Russian HCM patients, including age, sex ratio, family history of HCM, and SCD, did not differ. However, some distinctive features were found: G+ patients were diagnosed at the same age as other populations (around 38 years) [24], while G− patients were diagnosed with a significant delay compared to Western countries (around 57 versus 50 years) [9,24]. This may be attributed to the failure to recognize HCM in aged patients, often with comorbidities associated with secondary LV hypertrophy, and highlights the lack of awareness among specialists in Russia of a key feature of cardiomyopathies—age-dependent penetrance—and data on the role of comorbid conditions in the development of HCM, especially G− [6]. Russian HCM patients were underdiagnosed using family screening (10 vs. 16.6%) [28], which may reflect small family sizes and the distance between relatives. Other diagnostic procedures, except Holter monitoring, were underused, possibly due to a lack of accessibility and a relatively high cost. The rate of ICD implantation was extremely low (5.7 vs. 12–26%) [23,24,25,26,28], and the rate of SCD was about two times higher compared to Western populations [23,24,25]. The same low rate of ICD use (1% at enrollment) was reported in another Russian cohort [29]. The rate of all-cause mortality in a cohort of Russian HCM patients (2.86% per year) was also higher compared to studies from the last decade, and only similar to the SHaRe registry (2.76% per year for the whole cohort) [24], which has been enrolling patients since 1960, when the ICD implantation was not used. In contrast to ICD implantation, the rate of SRT (myectomy only) in Russia is high (21%), and similar to or even higher than in some Western populations [25,26]. This may be related to a high experience of Russian centers providing this type of care with good results [30]. Despite the significant proportion of symptomatic patients (80%), only 63% were treated with beta-blockers, which is lower than in other populations (74–81%) [23,28]. Furthermore, hypertensive patients received beta-blockers significantly more often than patients without hypertension. Taken together, this may indicate that beta-blockers were not often prescribed for HCM-related indications alone. A lower adherence to treatment in Russian patients cannot be excluded either.

In our cohort, we observed some sex-based differences in baseline characteristics that are consistent with previous studies [31]. Typically, females with HCM are diagnosed and recruited for research at a later age, which may explain the increased prevalence of symptoms and more advanced HF. Constitutionally smaller LV dimensions may predispose females to higher LVEF, potentially accounting for the increased prevalence of LVOTO. Prognosis in HCM also shows sex-specific differences [31], although data on which outcomes are influenced by sex are controversial. In our study, males and females had similar overall survival. However, females showed a trend towards the occurrence of AF and stroke. This trend has been observed in some previous studies [32], but not in most [31]. The study by Lakdawala et al. showed that the more pronounced age at initial diagnosis in females depended on G+ status and disappeared in G− patients [33]. We obtained the opposite result: the difference in age of onset was statistically significant in the G− group and not observed in G+ patients. Larger prospective studies are needed to elucidate the impact of sex and coexisting vital factors such as genotype on the clinical course of HCM.

The frequency of G+ status in our cohort was found to be 38%, with *MYBPC3* and *MYH7* being two prevalent genes accounting for three-quarters of G+ and one-third of all genotyped unrelated patients. This pattern mirrors similar findings in many other populations [7,24,26,34,35]. The types of variants (all missense for *MYH7* and with a prevalence of truncating for *MYBPC3*) were also consistent with previously reported data [20,21].

We identified 10 patients with multiple variants, representing 15% of G+ and 5.7% of all genotyped probands, including 4 subjects carrying only sarcomeric multiple variants (2.3% of all genotyped probands). These data are consistent with other populations where the prevalence of subjects with multiple variants ranges from 9% (1.7–2.7% of the genotyped cohort) [24,35,36] to 17–19% (5–6.7% of the genotyped cohort) [37,38], and depends on the number of genes analyzed and the criteria used for variant classification. Our conclusions regarding the clinical course of HCM patients with multiple variants are consistent with previous studies [24,35,36]. Despite the general trend toward a more severe phenotype, the combined effect of different double variants is highly variable and difficult to predict. Therefore, prognosis must be assessed on a case-by-case basis.

It has been noted that each population may have its own most common disease-associated variants, often with a founder effect [39,40,41]. The most common HCM causative variant in Western European populations is *p.R502W* in *MYBPC3*, which occurs in 1–7% of genotyped patients [24,26,34,42,43]. In our study cohort, the p.Q1233* variant in *MYBPC3* was the most common, detected in 4.5% of genotyped probands (12% of G+). All carriers of the p.Q1233* variant were from the Central (Moscow) District of Russia and of Slavic origin. The MYBPC3 p.Q1233* variant has also been found to be very common in some Eastern European countries, such as Belarus and Hungary [44], where its founder effect has been confirmed. In Western countries, the prevalence of the p.Q1233* variant is less than 1% [24,26,34]. In the only cited genetic study in Russian HCM population [29], the p.Q1233* variant was identified in 2% of genotyped patients, but the overall yield of genetic testing was unexpectedly low, only 20%, and the prevalence of the p.Q1233* variant among the G+ patients was 10%. Our results make the MYBPC3 p.Q1233* variant a candidate for a founder effect confirmation study. A larger study is needed to verify the regional clustering of the p.Q1233* variant in Russia.

While the truncating variants in *MYBPC3* cause HCM regardless of location, consistent with locus-independent loss of function, assessing the pathogenicity of non-truncating *MYBPC3* variants is challenging because not all are truly pathogenic. Missense variants in *MYBPC3* associated with HCM have been reported to be regionally clustered, and a subset also cause loss of function through failure of myofilament incorporation and rapid degradation [21]. Of the nine amino acid substitutions caused by *MYBPC3* missense variants found in our cohort, six are clustered in enriched domains 6 and 10 of MYBP-C. These domains are more likely to contain actionable variants [21], especially domain 10, which is considered a hotspot for HCM [20]. The *p.R346H* variant found in two of our probands is located in the tri-helix bundle (THB) portion of the M-domain of cardiac MYBP-C, which is not considered a hotspot region for *MYBPC3* variants. This variant has been previously reported in an individual with HCM, but without clinical data [34]. Another reported patient who died suddenly had an additional truncating variant in *MYBPC3*, and the *p.R346H* variant was classified as LB [45]. Current computational prediction tools support a deleterious effect of this variant on the gene, but segregation data are lacking, and despite several additional entries, ClinVar classifies the *p.R346H* variant as VUS. The THB is a specific motif for the cardiac isoform of MYBP-C, providing the platform for potential binding to actin and myosin [46]. In vitro studies have shown that the mutations in THB can alter these interactions [47]. Further evidence for the causality of the *p.R346H* variant was obtained by our family studies. All four carriers of the *p.R346H* variant had non-obstructive HCM with a benign clinical course.

Another variant found in two of our patients (1.1%), *p.A729P* in *MYH7*, is also suspected to have a founder effect in the Russian population. The *p.A729P* variant has only been identified in Russian [48] and Belarusian HCM patients [49] with a prevalence of 1.2–2.6%, and has not been reported in other populations.

Carriers of thin filament variants had a significantly lower LV wall thickness and a worse prognosis for HF, which is supported by previous studies [50]. There is strong evidence that thin filament causative variants contribute to Ca2+ dysregulation within the sarcomere, and may have a distinct disease pathogenesis from think-filament-associated HCM.

The *TPM1* gene is one of the major thin filament sarcomeric genes responsible for about 3–5% of all HCM cases [50], but clinical data on carriers are scarce, and evidence for the pathogenicity of *TPM1* variants is inconclusive. We described five affected individuals carrying the same *p.Q210R* variant and performed two family studies. The *p.Q210R* variant in *TPM1* was previously mentioned in a paper [51], but without clinical data. In our study, the *p.Q210R* variant segregated with a morphologically non-severe HCM phenotype and was associated with incomplete penetrance. Early fibrosis with signs of inflammation and arrhythmic risk may be a distinctive feature of this variant, but a larger study is needed.

Four probands and one relative from our cohort had disease-causing variants only in non-sarcomeric genes: *ALPK3* (2), *TRIM63* (1), and *FLNC* (2). Three of these patients have been described in previous studies [52,53,54]. This paper presents two patients with relatively benign clinical courses who were assigned to the G+ group based on carrying the single variants in *FLNC*. Filamin C protein maintains sarcomere integrity by crosslinking actin filaments at the sarcomeric Z-disc. It consists of 2 calponin homology domains, 24 immunoglobulin (Ig) domains divided into ROD1 and ROD2 subdomains, and a C-terminal dimerization domain [55]. Since the truncating variants in *FLNC* are highly enriched in an overlapping phenotype of dilated and left-dominant arrhythmogenic cardiomyopathy [56], missense variants, especially those located in the ROD2 domain, have recently been associated with HCM and restrictive cardiomyopathy phenotypes [22,57,58]. However, knowledge about the pathogenicity and genotype–phenotype correlations of missense *FLNC* variants remains scarce.

The genetic testing of the initial group identified four patients with other diseases unrelated to sarcomere dysfunction. Notably, all of these patients were over 60 years old, and three of them were undergoing specific treatment for transthyretin amyloidosis and Fabry disease. One of these cases has been described previously [59]. There are two important messages to be drawn from this: first, elderly patients often present with mild extracardiac features of systemic disease, or these symptoms may overlap with common age-related diseases and be overlooked by clinicians; second, genetic testing may be the only diagnostic tool to correctly diagnose rare diseases, especially in elderly patients.

The overall population of Russia, as well as each distinct ethnic group living on the territory of the Russian Federation, may potentially have their own ‘load of mutations’ [60] due to genetic drift, adaptation, or migration [61]. This can predetermine population-specific patterns of prevalence and traits of inherited diseases, including HCM, and may influence tailored clinical management specific to certain regions. To date, complete Russian genomes have not been sequenced for the presence and frequency of medically significant variants. However, the initial efforts to do this have revealed dramatic population stratification within the Russian population for some inherited traits, including differences in allele frequency that are important for medical issues [62]. Furthermore, studies on patients with inherited disorders have shown that Russians harbor unique causative genetic variants compared to other populations [63]. The few previous studies in underrepresented populations of HCM patients point to the need for genetic studies in different racial and ethnic groups. For example, a statistically significant reduction in the detection rate of P/LP variants for cardiomyopathy was observed in non-white individuals living in the USA [11]. This means that there are different spectrums of recurrent variants between different ethnicities, and variants from black people, for example, are less known because they are underrepresented in HCM research. There is also considerable diversity in the genetic architecture of HCM among European populations. Founder mutations contributing to a substantial proportion of cases have been identified in some regions with apparent population bottlenecks, such as the *MYBPC3* c.927-2A>G variant in Iceland with a population prevalence of 0.36% [64]; three Dutch founder mutations in *MYBPC3* in the Netherlands: c.2373_2374insG, c.2864_2865delCT, and c.2827C > T [65]; the *TPM1* p.Arg21Leu variant in Portugal and Spain; and many other studies [66]. This dictates region-specific studies of the genetics of HCM, because for some variants in certain regions, rarity will not be the main criterion for pathogenicity. The absence of the Russian ethnic background in control populations limits the current guidelines for variant interpretation.

A limitation of the study is the relatively small sample size of the study group which could impact the genetic spectrum of the disease and did not allow the acquisition of robust phenotype–genotype correlations. Also, the majority of the enrolled patients were of Slavic descent, so the generalizability of the findings to the broader Russian population, which comprises 195 ethnic groups, may not be correct. Additionally, the study may have a regional bias, as most patients originated from Moscow city. Mortality rates may be underestimated since only patients diagnosed during their lifetime were included.

## 5. Conclusions

Russian HCM patients share the same demographic and clinical characteristics as other HCM populations. Specific management and prognostic features have been identified that Russian clinicians need to be aware of. The genetic landscape of HCM in the Russian population generally replicates previous studies based on European ancestry, but the variant spectrum has some differences. Together with data from other understudied populations, this highlights the need to study the genetic background of HCM in different regions to enrich existing variant databases for a more accurate and efficient interpretation of variant pathogenicity. Based on our findings, a comprehensive study of HCM should be conducted across the entire territory of Russia, given its geographic and ethnic diversity.

## Figures and Tables

**Table 1:** Clinical characteristics with respect to sex in 193 Russian HCM patients.

Characteristics	Total Group *n* = 193	Males *n* = 100	Females *n* = 93	*p*-Value Males vs. Females
Demography				
Males/Females, *n* (%)	100 (52)/93 (48)	-	-	-
Age at enrollment, years (median [IQR])	56 [42–66]	50 [38–65]	60 [46–67]	0.011
Age at diagnosis, years (median [IQR])	49 [37–62]	45 [35–60]	54 [42–63]	0.018
Diagnosed over 60 years, *n* (%)	52 (27)	23 (23)	29 (31)	0.20
History of HCM				
Family history of HCM in probands, *n* (%)	44/176 (25)	21/92 (23)	23/84 (27)	0.51
Family history of SCD < 40 years ˆ, *n* (%)	22 (11)	4 (4)	18 (20)	0.001
Reason for diagnosis of HCM				
- Incidental, *n* (%)	56 (29)	37 (37)	19 (20)	0.011
- HCM-associated symptoms, *n* (%)	119 (62)	54 (54)	65 (70)	0.023
- Family screening, *n* (%)	17 (9)	9 (9)	9 (10)	0.87
First diagnosis at enrollment, *n* (%)	66 (34)	23 (23)	29 (31)	0.20
Asymptomatic at enrollment, *n* (%)	40 (21)	27 (27)	13 (14)	0.026
Obstructive HCM *, *n* (%)	93 (48)	46 (46)	47 (51)	0.53
5-year SCD risk score, %	2.7 [1.8–4.0]	3.2 [2.1–4.5]	2.4 [1.6–3.5]	0.002
5-year SCD risk score > 6%, *n* (%)	22 (11)	15 (17)	7 (8)	0.07
HCM-associated events in past history				
NYHA class III/IV, *n* (%)	43 (22)	16 (16)	27 (29)	0.030
Ventricular tachycardia, *n* (%)	34 (18)	20 (20)	14 (15)	0.24
Atrial fibrillation, *n* (%)	52 (27)	21 (21)	31 (33)	0.05
Stroke/TIA, *n* (%)	11 (6)	7 (7)	4 (4)	0.42
Hypokinetic HCM, *n* (%)	8 (4)	3 (3)	5 (5)	0.49
Comorbidities				
Arterial hypertension, *n* (%)	121 (63)	62 (62)	59 (63)	0.84
Obesity (BMI ≥ 30 kg/m^2^), *n* (%)	60 (31)	32 (32)	28 (30)	0.78
Documented CAD **, *n* (%)	19 (10)	8 (8)	11 (12)	0.37
Diabetes mellitus, *n* (%)	22 (11)	7 (7)	15 (8)	0.05
Diagnostics at enrollment				
24 h Holter monitoring, *n* (%)	150 (78)	76 (76)	74 (80)	0.55
Contrast CMR, *n* (%)	70 (36)	38 (38)	32 (34)	0.60
- LGE, *n* (%)	54/70 (77)	30/38 (79)	24/32 (75)	0.70
- Apical aneurysm, *n* (%)	2/70 (3)	1/38 (2.6)	1/34 (3)	0.99
- Non-compaction myocardium, *n* (%)	4/70 (6)	3/38 (8)	1/34 (3)	0.62
(CT) coronary angiography, *n* (%)	89 (46)	42 (42)	47 (51)	0.23
Stress Echocardiography, *n* (%)	25 (13)	14 (14)	11 (12)	0.65
NT-proBNP, pg/mL (median [IQR])	816 [260–2102]	585 [197–1737]	917 [463–2567]	0.06
Creatinine, µmoL/l (median [IQR])	92 [76–106]	96 [86–112]	78 [69–98]	<0.0001
Treatment at enrollment and during follow-up				
Beta-blockers, *n* (%)	122 (63)	61 (62)	61 (66)	0.57
ICD implantation, *n* (%)	11 (5.7)	3 (3)	8 (8.6)	0.09
Septal reduction therapy, *n* (%)	36 (19)	17 (17)	19 (20)	0.54
Alcohol septal ablation, *n* (%)	4 (2)	3 (3)	1 (1)	0.62
Mitral valve surgery, *n* (%)	12 (6)	3 (3)	9 (9.7)	0.06
Pacemaker, *n* (%)	10 (5)	2 (2)	8 (8.6)	0.05
- Not related to SRT, *n* (%)	6 (3)	0	6 (6.5)	0.011
Atrial fibrillation/flutter ablation, *n* (%)	9 (4.7)	4 (4)	5 (5)	0.74

HCM—hypertrophic cardiomyopathy; SCD—sudden cardiac death; NYHA—New York Heart Association; TIA—transient ischemic attack; BMI—body mass index; CAD—coronary artery disease; CMR—cardiac magnetic resonance; LGE—late gadolinium enhancement; CT—computed tomography; NT-proBNP—N-terminal pro brain natriuretic peptide; ICD—implantable cardioverter-defibrillator; SRT—septal reduction therapy; AF—atrial fibrillation; HF—heart failure. ˆ First- and second-degree relatives. * LV outflow gradient ≥ 30 mmHg at rest or after provocation. ** Myocardial infarction or revascularization or significant (>50%) coronary atherosclerosis.

**Table 2:** Data of echocardiography, electrocardiography, and Holter monitoring with respect to sex in 193 Russian HCM patients.

	Total Group *n* = 193	Males *n* = 100	Females *n* = 93	*p*-Value Males vs. Females
Echocardiography (100% of included patients)				
Morphology				
Apical HCM, *n* (%)	25 (13)	12 (12)	13 (14)	0.68
Midventricular HCM, *n* (%)	5 (2.6)	2 (2)	3 (3)	0.67
Maximal LVWT, mm (median [IQR])	20 [17.5–23]	20 [18–24]	20 [17–22]	0.042
Indexed maximal LVWT, mm/m^2^ (median [IQR])	10 [9–12]	10 [8–12]	11 [10–13]	0.025
Extreme LVH (≥30 mm), *n* (%)	6 (3)	5 (5)	1 (1)	0.21
Non-compaction myocardium, *n* (%)	8 (4)	6 (6)	2 (2)	0.18
Indexed LV EDV, mL/m^2^ (median [IQR])	46.5 [35–56]	49 [42–59]	43 [32–53]	0.001
Indexed LV ESV, mL/m^2^ (median [IQR])	15 [10–20]	17 [12–21]	12 [9–18]	0.001
Indexed LV EDV < 50 mL/m^2^, *n* (%)	118 (61)	54 (54)	64 (69)	0.035
LA diameter, mm (median [IQR])	44 [40–49]	46 [41–51]	43 [39–46]	0.025
LA diameter ≥ 45 mm, *n* (%)	91 (47)	54 (54)	37 (40)	0.05
Indexed LA ESV, mL/m^2^	42 [33–53]	41 [33–50]	42 [33–56]	0.42
**Functional parameters**				
LVOT gradient at rest, mmHg	14 [5–55]	13 [5–45]	15 [7–66.5]	0.18
LVOTO ≥ 30 mmHg at rest, *n* (%)	61 (32)	30 (30)	31 (33)	0.62
LVOTO ≥ 30 mmHg after provocation (latent), *n* (%)	32 (17)	15 (15)	17 (18)	0.56
Indexed LV stroke volume, mL/m^2^ (median [IQR])	30 [25–38]	32 [27–39]	29 [23–34]	0.013
Indexed LV stroke volume < 30 mL/m^2^, *n* (%)	101 (52)	44 (44)	57 (61)	0.016
LVEF, % (median [IQR])	67 [60–73]	65 [59–71]	69 [62–73]	0.020
- LV diastolic dysfunction, *n* (%)	162 (84)	81 (81)	81 (87)	0.25
Restrictive type, *n* (%)	20 (10)	12 (12)	8 (8.6)	0.44
E/e’	11 [7–17]	9 [6–17]	12 [8–17]	0.007
SPAP > 40 mmHg, *n* (%)	35 (18)	14 (14)	21 (23)	0.13
Electrocardiography (100% of included patients)				
Normal electrocardiogram, *n* (%)	4 (2.1)	2 (2.0)	2 (2.2)	0.94
Sinus rhythm, *n* (%)	181 (94)	96 (96)	85 (91)	0.19
Pathological Q waves, *n* (%)	43 (22)	20 (20)	23 (25)	0.45
Poor R wave progression in V1-V3(V4), *n* (%)	48 (25)	22 (22)	26 (28)	0.36
T wave inversion *, *n* (%)	141 (73)	76 (76)	65 (70)	0.34
Positive T wave in aVR, *n* (%)	85 (44)	47 (48)	38 (41)	0.36
Sokolow–Lyon index, mm (median [IQR])	30 [21–41]	30 [24–42]	30 [20–41]	0.45
Sokolow–Lyon index ≥ 35 mm, *n* (%)	75 (39)	38 (38)	37 (40)	0.80
Left anterior fascicular block, *n* (%)	30 (16)	14 (14)	16 (17)	0.54
Complete LBBB, *n* (%)	11 (5.7)	6 (6)	5 (5)	0.80
Complete RBBB, *n* (%)	7 (3.6)	1 (1)	6 (6.5)	0.12
AV-block 1st degree, *n* (%)	24 (12)	18 (18)	6 (6.5)	0.010
24 h Holter monitoring (78% of included patients)				
NSVT, *n* (%)	29/150 (19)	16/76 (21)	13/74 (18)	0.59
VPBs > 500, *n* (%)	24/150 (16)	13/76 (17)	11/74 (15)	0.66
SVT / SPBs > 500, *n* (%)	57/150 (38)	26/76 (34)	31/74 (42)	0.44
Conduct disturbance **, *n* (%)	41/150 (27)	25/76 (33)	16/74 (22)	0.10

HCM—hypertrophic cardiomyopathy; LVWT—left ventricular wall thickness; LVH—left ventricular hypertrophy; EDV—end-diastolic volume; ESV—end-systolic volume; LA—left atrial; LV – left ventricle; LVOTO—left ventricular outflow tract obstruction; LVEF—left ventricular ejection fraction; E/e’—early transmitral flow velocity to early mitral annular tissue velocity to estimate LV filling pressure; SPAP—systolic pulmonary artery pressure; LBBB—left bundle branch block; RBBB—right bundle branch block; AV—atrioventricular; NSVT—non-sustained ventricular tachycardia; VPBs—ventricular premature beats; SVT—supraventricular tachycardia; SPBs—supraventricular premature beats. * ≥1 mm in ≥2 contiguous leads except leads aVR and V1. ** Sinoatrial- and AV-blocks.

**Table 3:** Genetic findings in 176 Russian unrelated HCM patients.

	All Probands *n* = 176
Genotype-positive subjects, *n* (%)	66 (38)
Sarcomere-positive subjects, *n* (%)	62 (35)
VUS only subjects, *n* (%)	7 (4)
At least one P/LP/VUS-LP variant, *n* (%)	
MYBPC3	31 (18)
- truncating/non-truncating	21/10 (12/5.7)
- p.Q1233*	8 (4.6)
MYH7	20 (11)
TPM1	6 (3.4)
TNNT2	2 (1)
TNNI3	2 (1)
MYL2	1 (0.6)
MYL3	0
ACTC1	1 (0.6)
TNNC1	2 (1)
FLNC	5 (2.8)
- FLNC only	1/6 (0.6)
ALPK3	2/37 (5)
TRIM63	1/50 (2)
Multiple P/LP/VUS-LP variants, *n* (%)	10 (5.7)
- Sarcomeric multiple variants only	4 (2.3)

P—pathogenic; LP—likely pathogenic; VUS—variant of uncertain significance; MYBPC3—cardiac myosin-binding protein C; MYH7—myosin heavy chain; TPM1—tropomyosin; TNNT2—cardiac troponin T; TNNI3—cardiac troponin I; MYL2—regulatory myosin light chain; MYL3—essential myosin light chain; ACTC1—alpha-actin; TNNC1—troponin C; FLNC—filamin C; ALPK3—Alpha-protein kinase 3; TRIM63—tripartite motif containing 63.

**Table 4:** Differences between genotype-positive and genotype-negative HCM patients (statistically significant variables only).

	G+ Group *n* = 78	G− Group *n* = 115	*p*-Value
Age at enrollment, years (median [IQR])	45 [35–56]	61 [50–68]	<0.0001
Age at diagnosis, years (median [IQR])	38 [25–47]	57 [47–66]	<0.0001
- Males/Females, (median [IQR])	36 [25–46]/ 41 [28–51]	50 [41–66]/ 60 [53–67]	0.33 for G+ group 0.016 for G− group
Diagnosed over 60 years, *n* (%)	9 (12)	43 (37)	<0.0001
Family history of HCM in probands, *n* (%)	44 (56)	17 (15)	<0.0001
Reason for diagnosis of HCM			
- Incidental, *n* (%)	32 (41)	24 (21)	0.002
- HCM-associated symptoms, *n* (%)	34 (44)	85 (74)	<0.0001
- Family screening, *n* (%)	12 (15)	6 (5)	0.017
Asymptomatic at enrollment, *n* (%)	26 (33)	14 (12)	<0.0001
5-year SCD risk score, %	3.1 [2.0–4.5]	2.5 [1.7–3.6]	0.050
5-year SCD risk score > 6%, *n* (%)	13 (19)	9 (9)	0.040
NYHA class III/IV, *n* (%)	10 (13)	33 (29)	0.009
Atrial fibrillation, *n* (%)	14 (18)	38 (33)	0.020
Arterial hypertension, *n* (%)	27 (35)	94 (82)	<0.0001
Obesity, *n* (%)	17 (22)	43 (37)	0.022
Documented CAD, *n* (%)	2 (3)	17 (15)	0.005
Contrast CMR, *n* (%)	35 (45)	35 (30)	0.031
LGE, *n* (%)	33 (83)	21 (51)	0.003
Beta-blockers, *n* (%)	41 (53)	81 (71)	0.009
Non-compaction myocardium, *n* (%)	9 (12)	2 (1.7)	0.008
LVOTO ≥ 30 mmHg at rest, *n* (%)	18 (23)	43 (37)	0.036
LV diastolic dysfunction, *n* (%)	59 (76)	103 (90)	0.010
Positive T wave in aVR, *n* (%)	27 (35)	58 (50)	0.036
Sokolow–Lyon index ≥ 35 mm, *n* (%)	17 (22)	58 (50)	<0.0001

HCM—hypertrophic cardiomyopathy; SCD—sudden cardiac death; NYHA—New York Heart Association; CAD—coronary artery disease; CMR—cardiac magnetic resonance; LGE—late gadolinium enhancement; LV—left ventricle; LVOTO—left ventricular outflow tract obstruction.

**Table 5:** Summary of outcomes and comparison of mortality to other populations.

	*n* (% of Total Group)/Incidence Rate (%) per Year			
	Our Cohort	Portuguese Registry [19]	SHaRe Registry [20]	UK, Spain, Greece, Italy [21]
Total number of patients	193	1042	4591	4893
Median follow-up, years	2.8	5.3	2.9	6.2
All-cause death	16 (8)/2.86	65 (6)/1.19	370 (8)/2.76	721 (14.7)/2.37
- Sudden cardiac death	5 (2.6)/0.93	12 (1.2)/0.22	58 (1)/0.34	168 (3.4)/0.55
New onset AF and stroke	18 (9)/3.21	-	-	-
- Males	4 (4)/1.43			
- Females	14 (15)/5.36			
HF outcome	15 (7.8)/2.79	-	-	-
- Thin filament carriers	3 (20)/7.14			
- Thin filament non-carriers	3 (4.8)/1.71			
Composite outcome	42 (22)/7.86	-	-	-

AF–atrial fibrillation; HF–heart failure.

## Data Availability

The data that support the findings of this study are available within the manuscript.

## Supplementary Materials

The following are available online at https://www.mdpi.com/article/10.3390/genes14112042/s1, Table S1: Clinical characteristics of Russian HCM patients with genetic findings.
