# Supplementary material for: Hypertrophic Cardiomyopathy in Underrepresented Populations: Clinical and Genetic Landscape Based on a Russian Single-Center Cohort Study
Source: Genes (Basel). 2023 Nov 4;14(11):2042. doi: 10.3390/genes14112042 (PMC10671745; doi:10.3390/genes14112042)
Supplement: Supplementary file 1 [file genes-14-02042-s001.zip › genes-2645868-supplementary.pdf]

Table S1. Clinical characteristics of Russian HCM patients with genetic findings (G+ patients are coloured in grey)

ID*	Sex	Age at evaluation (year)	Genotype			Age at initial diagnosis (year)	LVH pattern / max LVWT (mm)	LVOTO	Distinct features	Outcomes
			Gene	Variant**	Variant classification (→ reassessed)					
MYBPC3 CARRIERS										
1S	M	58	MYBPC3	**NM_000256.3:c.971del:p.I324Tfs*26**	P/LP	58	AS / 19	No	Severe AH	No
2S	F	36	MYBPC3	NM_000256.3:c.927-9G>A	P/LP	36	AS / 23	Yes	Asymptomatic	AF, embolic stroke, ICD
5B	F	61	MYBPC3	NM_000256.3:c.1273C>T:p.Q425*	P/LP	43	AS / 16	Yes	VT, conduction abnormalities	Hypokinetic HCM, AF, ICD
7S	F	29	MYBPC3	NM_000256.3:c.1351+2T>C	P/LP	26	AS / 16	Yes	Elite athlete	No
8S	F	45	MYBPC3	NM_000256.3:c.3697C>T:p.Q1233*	P/LP	36	AS / 15	Yes	Mild symptomatic 3 deliveries	Myectomy at 51
9E	M	34	MYBPC3	NM_000256.3:c.3697C>T:p.Q1233*	P/LP	34	AS / 17	No	Asymptomatic	No
12A	F	62	MYBPC3	NM_000256.3:c.2441_2443del:p.K814del	P/LP	45	AS / 24	Yes	SVA, severe AH	No
14H	F	34	MYBPC3	NM_000256.3:c.3697C>T:p.Q1233*	P/LP	23	AS / 18	Yes	VT, multiple SCD in family	Myectomy at 26, ICD at 34, AF

17K	F	65	MYBPC3	NM_000256.3:c.1120C>T:p.Q374*	P/LP	64	Ap / 20	No	CAD, SVA	AF, syncope
20K[1]	M	61	MYBPC3	NM_000256.3:c.624G>C:p.Q208H	VUS→VUS	60	Ap-AS / 20	Yes	Syncope	Myectomy at 67
26K	M	46	MYBPC3	NM_000256.3:c.2738-1G>A	P/LP	34	Ap / 26	No	Asymptomatic, VT	No
31S	M	33	MYBPC3	NM_000256.3:c.966G>A:p.W322*	P/LP	21	AS / 23	Yes	NYHA II, angina	No
32T	M	67	MYBPC3	NM_000256.3:c.3811C>T:p.R1271*	P/LP	47	AS / 21	No	VT, VEx, Cond abnormalities	Alive
33E	M	38	MYBPC3	NM_000256.3: c.3407_3409del:p.Y1136del	P/LP	38	AS / 20	Yes	NC, asymptomatic	No
37G	M	45	MYBPC3	NM_000256.3:c.3697C>T:p.Q1233*	P/LP	44	AS / 39	Yes	NYHA II, SVA	No
41D[2]	F	44	MYBPC3	**NM_000256.3: c.2781_2782insCACA: p.S928Hfs*124**	P/LP	37	AS / 23	Yes	NYHA III	Myectomy at 46
41Da[2] Sib-twin	M	44	MYBPC3	NM_000256.3: c.2781_2782insCACA: p.S928Hfs*124	P/LP	44	AS / 16	No	II NYHA	No
42P	M	34	MYPBC3	NM_000256.3:c.1037G>A:p.R346H	VUS→VUS-LP	21	Ap / 26	No	Asymptomatic	No
42Pa Father	M	60	MYBPC3	NM_000256.3:c.1037G>A:p.R346H	VUS→VUS-LP	27	Ap / 21	No	NYHA II	No
51K	M	35	MYBPC3	NM_000256.3:c.3697C>T:p.Q1233*	P/LP	16	AS / 26	No	VEx, VT	ICD at 37

51Ka Sib	M	38	MYBPC3	NM_000256.3:c.3697C>T:p.Q1233*	P/LP	17	AS / 21	No	SVA	ICD at 39, AF
60L	F	62	MYBPC3	NM_000256.3:c.2429G>A:p.R810H	P/LP	61	AS / 24	No	NYHA III, Cond abnormalities	Hypokinetic HCM, AF
65K	M	60	MYBPC3	NM_000256.3:c.1731G>A:p.W577*	P/LP	47	AS / 23	Yes	VT, 1st AV-block	Myectomy at 60
70L	M	51	MYBPC3	NM_000256.3:c.3697C>T:p.Q1233*	P/LP	45	AS / 25	Yes	Syncope, NYHA II	No
75M[2]	F	29	MYBPC3	NM_000256.3:c.3697C>T:p.Q1233*	P/LP	17	AS / 26	No	NYHA II	No
**MYH7 CARRIERS**										
11L	F	56	MYH7	NM_000257.4:c.5135G>A:p.R1712Q	P/LP	51	AS / 25	No	NYHA II, Cond abnormalities	No
18L	M	54	MYH7	NM_000257.4:c.2185G>C:p.A729P	P/LP	36	AS / 18	No	Angina, VT	No
23B	M	36	MYH7	NM_000257.4:c.2276G>A:p.G759D	VUS→P/LP	24	Ap / 18	No	NC	No
24H	M	36	MYH7	NM_000257.4:c.2129C>T:p.P710L	P/LP	24	AS / 17	Yes	Asymptomatic, VT	No
34P	F	45	MYH7	NM_000257.4:c.2185G>C:p.A729P	P/LP	40	AS / 18	No	Asymptomatic	No
35N	M	52	MYH7	NM_000257.4: c.5134C>T:p.R1712W	P/LP	47	AS / 18	Yes	Syncope, NYHA III	Myectomy at 56, AF
36S	F	64	MYH7	NM_000257.4:c.3569C>T:p.A1190V	P/LP	62	Ap / 24	No	Angina, SVA	No

43S	F	62	MYH7	NM_000257.4:c.1615A>G:p.M539V	P/LP	42	/ 16	No	NYHA III, Sinus arrest, restrictive DD	Hypokinetic HCM, AF, Pacemaker
44S	F	60	MYH7	NM_000257.4:c.3652G>C:p.E1218Q	P/LP	57	AS / 19	Yes	NYHA III, Cond abnormalities	Myectomy at 62, AF, pacemaker
45F	F	67	MYH7	NM_000257.4:c.1711G>A:p.G571R	VUS→P/LP	66	SC / 22	Yes	NYHA III, severe AH	Myectomy at 67, AF
46S	M	36	MYH7	NM_000257.4:c.632C>T:p.P211L	P/LP	33	AS / 23	Yes	Asymptomatic	No
48R[2]	M	52	MYH7	NM_000257.4:c.746G>A:p.R249Q	P/LP	32	AS / 22	No	NYHA II, SVA	AF
48Ra[2] Daughter	F	21	MYH7	NM_000257.4:c.746G>A:p.R249Q	P/LP	8	AS / 28	No	NYHA II Delivery	No
53M	F	54	MYH7	**NM_000257.4:c.4163A>G:p.E1388G**	VUS→VUS-LP	54	AS / 18	No	Severe AH, NYHA II	No
72A	M	16	MYH7	NM_000257.4:c.2221G>A:p.G741R	P/LP	2	AS / 23	Yes	NYHA III	Myectomy at 6
72Aa mother	F	41	MYH7	NM_000257.4:c.2221G>A:p.G741R	P/LP	41	PM / 13	No	Asymptomatic	No
73A[2]	M	38	MYH7	NM_000257.4:c.2360G>A:p.R787H	VUS→VUS	31	Ap-AS / 43	No	NYHA II, angina, SVA, presyncope	No
76P	F	25	MYH7	NM_000257.4:c.1208G>A:p.R403Q	P/LP	15	AS / 30	Yes	NYHA II, syncope	No
77N	F	44	MYH7	NM_000257.4:c.2791G>A:p.E931K	P/LP	13	AS / 18	No	NYHA II, VT, SVA	No
OTHER SARCOMERE CARRIERS										
29S	F	41	TPM1	NM_001018005.2:c.629A>G:p.Q210R	P/LP	29	AS / 17	No	Mild hydropericardi	No

									um, VT asymptomatic	
30K	F	57	TPM1	NM_001018005.2:c.629A>G:p.Q210R	P/LP	44	Meso / 22	Yes	1st AV-block	No
30Ka sib	F	49	TPM1	NM_001018005.2:c.629A>G:p.Q210R	P/LP	49	AS / 13	No	Asymptomatic	No
39S	M	68	TPM1	NM_001018005.2:c.629A>G:p.Q210R	P/LP	68	AS / 19	No	Asymptomatic	No
39Sa daughter	F	38	TPM1	NM_001018005.2:c.629A>G:p.Q210R	P/LP	37	AS / 13	No	NYHA II, mild hydropericardi um	No
63A	M	48	TPM1	**NM_001018005.2:c.86A>G:p.K29R**	VUS→VUS-LP	42	AS / 25	No	Asymptomatic	No
66C	F	32	TPM1	NM_001018005.2:c.574G>A:p. p.E192K	P/LP	32	AS / 20	No	Asymptomatic, SVT	No
21U	F	56	TNNT2	NM_001276345.2:c.341C>T:p.A114V	P/LP	48	AS / 20	Yes	NYHA II, Cond abnormalities, angina	No
25C	M	67	TNNT2	NM_001276345.2:c.862C>T:p.R288C	P/LP	67	AS / 27	Yes	Mild Ao stenosis	Stroke at 66
6E	F	50	TNNI3	NM_000363.5:c.557G>A:p.R186Q	P/LP	36	Meso / 18	No	RV obstruction	AF, SVA, NYHA III
56H[3]	M	25	TNNI3	NM_000363.5:c.422G>A:p.R140Q	P/LP	25	Ap / 17	No	Asymptomatic	No
10G	F	28	MYL2	NM_000432.4:c.275-8C>A	VUS→LP	5	Bi, Meso / 23	Yes	Restrictive DD, NYHA II, Cond abnormalities	AF, ICD at 36
22S	F	44	MYL2	NM_000432.4:c.353+7G>A	VUS→VUS	28	AS / 25	No	SVA, VT	ICD at 44

NON-SARCOMERE CARRIERS										
38V	M	38	ALPK3	NM_020778.5:c.4689delG:p.W1563*	P/LP	34	SC / 32	Yes	Severe AH, VT	Myectomy at 38
38Va Father	M	62	ALPK3	NM_020778.5:c.4689delG:p.W1563*	P/LP	62	AS / 28	Yes	Severe AH, Asymptomatic	No
38Vb Sib	F	36	ALPK3	NM_020778.5:c.4689delG:p.W1563*	P/LP	36	AS / 13	No	Asymptomatic	No
4Ga son	M	42	FLNC	NM_001458.5:c.5500C>T:p.H1834Y	VUS→VUS-LP	42	AS / 13	No	Asymptomatic	No
28M[2]	F	71	FLNC	NM_001458.5:c.5945G>A:p.R1982H	VUS→VUS-LP	71	Ap / 20	No	NYHA III, VEx	No
55M	M	67	FLNC	NM_001458.5:c.2635C>T:p.R879C	VUS→VUS-LB	67	AS / 20	Yes	CAD, NYHA II	AF
16N	M	77	FHOD3	NM_001281740.3:c.4375C>T:p.R1459W	VUS→VUS-LB	40	AS / 9	No	VT, LVEF 35%	Hypokinetic HCM, death from HF
61L	M	41	FHOD3	**NM_001281740.3:c.2429G>A:p.G810E**	VUS→VUS	41	Ap / 16	No	Asymptomatic, Cond abnormalities	No
69R	F	51	TCAP	NM 003673.4:c.448G>A:p.G150S	VUS→VUS	51	AS / 16	No	Myalgia, CK elevation, angina, NYHA III	No
74A[2]	M	42	BAG3	**NM_004281.4:c.656C>T:p.P219L**	VUS→VUS	41	Ap-A / 24	No	Angina	No
49D	M	66	GAA	NM_000152.5:c.2237G>C:p.W746S	VUS→VUS	64	Meso / 23	No	NYHA II, VEx, SVA, midvent obstruction	No
59D	M	69	LAMP2	**NM_002294.3:c.244delG:p.D82Ifs*7** (Mosaicism 17%)	P/LP	69	AS / 17	Yes	VT, SVA	No

MULTIPLE VARIANTS CARRIERS										
15M	M	18	TRIM63<br>TRIM63	NM_032588.4:c.739C>T:p.Q247*<br>NM_032588.4:c.224G>A:p.C75Y	P/LP	15	AS / 24	No	Asymptomatic athlete	No
3B	F	29	MYBPC3<br>TNNC1	NM_000256.3:c.3697C>T:p.Q1233*<br>NM_003280.3:c.86T>A:p.L29Q	P/LP<br>P/LP	22	AS / 25	Yes	3 deliveries, 4 children	Myectomy at 28, SCD at 42
4G	F	51	MYBPC3<br>FLNC	**NM_000256.3:c.3790T>C:p.C1264R**<br>NM_001458.5:c.5500C>T:p.H1834Y	P/LP<br>VUS→VUS-LP	51	SC / 20	Yes	Severe AH, SCD of eldest son	Hypokinetic HCM, AF, fatal stroke at 57
19B	F	33	MYBPC3<br>FLNC	NM_000256.3:c.743_746del: p.D248Afs*51<br>NM_001458.5:c.4413A>T: p.Q1471H	P/LP<br>VUS→VUS-LB	19	Ap-AS / 22	No	NYHA II	No
27K	M	46	MYBPC3<br>FLNC	NM_000256.3:c.2905+1G>A<br>NM_001458.5:c.1609T>G:p.Y537D	P/LP<br>VUS→LP	34	AS / 26	No	NYHA III	AF
47S	M	60	MYH7<br>FLNC	NM_000257.4:c.4180G>A:p.A1394T<br>NM_001458.5:c.6214G>A:p.G2072R	VUS→VUS-LP<br>VUS→VUS-LP	60	AS / 18	No	Expanded fibrosis (41% LGE)	AF
50S	F	61	TPM1<br>MYBPC3	**NM_001018005.2:c.74C>T:p.A25V**<br>NM_000256.3:c.932C>T: p.S311L	P/LP<br>VUS→VUS	61	Ap-AS / 14	No	Asymptomatic	No
50Sa Sib	M	55	TPM1<br>MYBPC3	NM_001018005.2:c.74C>T:p.A25V<br>NM_000256.3:c.932C>T: p.S311L	P/LP<br>VUS→VUS	55	Ap-AS / 15	No	NYHA II	No
52D[2]	M	24	MYH7<br>FHOD3	NM_000257.4:c.2156G>A:p.R719Q	P/LP	23	AS / 15	No	NC, LVEF < 65%	AF, PV ablation

				NM_001281740.3:c.1754C>A:p.S585Y	VUS→VUS-LB					
54V	M	51	MYBPC3<br>MYBPC3	NM_000256.3:c.3794A>T:p.E1265V<br>NM_000256.3:c.3796T>C:p.C1266R	P/LP<br>P/LP	40	AS / 23	No	SVA	AF
62B	M	39	MYBPC3<br>ALPK3	NM_000256.3:c.1037G>A:p.R346H<br>NM_020778.5:c.3967G>A:p.A1323T	VUS→VUS-LP<br>VUS→VUS	39	Ap-AS / 17	No	Asymptomatic ST elevation	No
62Ba Sib	M	33	MYBPC3<br>ALPK3	NM_000256.3:c.1037G>A:p.R346H<br>NM_020778.5:c.3967G>A:p.A1323T	VUS→VUS-LP<br>VUS→VUS	30	AS / 18	No	Cond abnormalities ST elevation	No
64B	F	46	ACTC1<br>FLNC	NM_005159.5:c.940C>T:p.R314C<br>**NM_001458.5:c.15C>A p.5S>R**	P/LP<br>VUS-LB	46	AS / 16	Yes	SVA, Cond abnormalities	Alcohol ablation
67V	M	51	MYBPC3<br>GLA	NM_000256.3:c.2623C>T:p.H875Y<br>NM_000169.3:c.1060A>G:p.I354V	VUS→VUS-LP<br>VUS→LB	51	AS / 21	Yes	SVA	AF, Myectomy at 51
68A	M	35	TNNC1<br>MYH7	NM_003280.3:c.435C>A:p.D145E<br>**NM_000257.4:c.3627C>G:p.N1209K**	P/LP<br>VUS→VUS-LP	35	AS / 16	Yes	Angina, NYHA II	No
71Z	M	47	FLNC<br>FLNC	NM_001458.5:c.2377G>A:p.E793K<br>NM_001458.5:c.5020G>A:p.G1674S	VUS→VUS-LB<br>VUS→VUS-LB	47	AS / 15	No	Angina	No
78K	F	42	MYBPC3<br>MYH7	NM_000256.3:c.3697C>T:p.Q1233*<br>NM_000257.4:c.5137G>A:p.V1713M	P/LP<br>VUS→VUS	42	PM / 17	No	Palpitations Severe HCM in child (same genotype)	No
13S	M	40	MYBPC3	NM_000256.3:c.3763G>A:p.A1255T	P/LP	39	AS / 25	Yes	NC, VT, VEx, SVEx	Death from HF at 44

			MYBPC3<br>FLNC	NM_000256.3:c.538G>A:p.G180S<br>NM_001458.5:c5500C>T:p.H1834Y	VUS→VUS<br>VUS→VUS-LP					
40R	M	29	MYH7<br>MYH7<br>PTPN11	**NM_000257.4:c.137T>A:p.F46Y**<br>NM_000257.4:c.5346G>A: p.M1782I<br>NM_001330437.1: c.925A>G:p.I309V	VUS→VUS-LP<br>VUS→LP<br>VUS→LB	14	AS / 19	Yes	NC, Cond abnormalities	Myectomy at 31
58M[2]	M	38	ALPK3<br>ALPK3<br>MYBPC3	NM_020778.5:c.1958C>G:p.S653*<br>NM_020778.5:c.3491G>A:p.R1164Q<br>NM_000256.3:c.667G>A:p.E223K	P/LP<br>VUS→VUS-LP<br>VUS→VUS	38	SC / 16	No	Restrictive DD, LVEF 34%	Hypokinetic HCM

A – anterior; AH – arterial hypertension; Ao – aorta; Ap – apical; AS – asymmetric septal; Bi – biventricular hypertrophy; CK – creatine kinase; Cond – conduction (abnormalities); DD – diastolic dysfunction; ID – identification number of patients in the present study; LGE – late gadolinium enhancement; Meso – mesoventricular; NC – non-compaction myocardium; NYHA - New York Heart Association; PM – papillary muscle; SC – symmetric concentric; Sib – sibling; SVEx – supraventricular extrasystole; SVA – supraventricular arrhythmia; VEx – ventricular extrasystole (> 500/24h); VT – ventricular tachycardia;

*in order of inclusion in the study

**Novel variants are spelled in bold; All variants are in heterozygosity

[1]Jewdish origin; [2]Turkic origin; [3]Armenian origin
